# Three-dimensional Imaging of Crystalline Inclusions Embedded in Intact Maize Stalks

**DOI:** 10.1038/srep02843

**Published:** 2013-10-03

**Authors:** John Badger, Jyotsana Lal, Ross Harder, Hideyo Inouye, S. Charlotte Gleber, Stefan Vogt, Ian Robinson, Lee Makowski

**Affiliations:** 1 DeltaG Technologies, San Diego, California 92122, USA; 2 X-ray Science Division, Advanced Photon Source, Argonne National Laboratory, Argonne, Illinois 60439, USA; 3 Dept. of Electrical and Computer Engineering, Northeastern University, Boston, Massachusetts 02115, USA; 4 London Center for Nanotechnology, University College London, London WC1E 6BT, UK; 5 Research Complex at Harwell, Oxford, OX11 0FA, UK

## Abstract

Mineral inclusions in biomass are attracting increased scrutiny due to their potential impact on processing methods designed to provide renewable feedstocks for the production of chemicals and fuels. These inclusions are often sculpted by the plant into shapes required to support functional roles that include the storage of specific elements, strengthening of the plant structure, and providing a defense against pathogens and herbivores. *In situ* characterization of these inclusions faces substantial challenges since they are embedded in an opaque, complex polymeric matrix. Here we describe the use of Bragg coherent diffraction imaging (BCDI) to study mineral inclusions within intact maize stalks. Three-dimensional BCDI data sets were collected and used to reconstruct images of mineral inclusions at 50–100 nm resolution. Asymmetries in the intensity distributions around the Bragg peaks provided detailed information about the deformation fields within these crystal particles revealing lattice defects that result in distinct internal crystal domains.

Characterization of a heterogeneous sample such as corn stover involves dealing with material that is organized on multiple length scales and contains discrete domains with different compositions, orientations and order. Investigations into the structural components of these types of sample are of considerable interest since the presence of mineral inclusions in plant tissues is a factor in the development of industrial processes used to produce chemicals and fuel^1^. In addition, the inclusion of crystals in plant tissues has been identified with several distinct biological functions that include providing sinks for chemical accumulation, adding structural support to plant tissues and creating a defense against pathogens and herbivores^2^,^3^,^4^.

A novel approach for non-destructive *in situ* imaging of the sizes and morphologies of micro crystals embedded in opaque media is provided by Bragg coherent diffraction imaging (BCDI). This approach has previously been used to characterize manufactured samples of simple crystals^5^,^6^,^7^,^8^. Here we demonstrate the applicability of BCDI to more complex crystals embedded in biological samples and, more generally, the use of the technique for analyzing sample types in which crystalline particles are concealed within an amorphous media.

## Results

**Figure 1:**
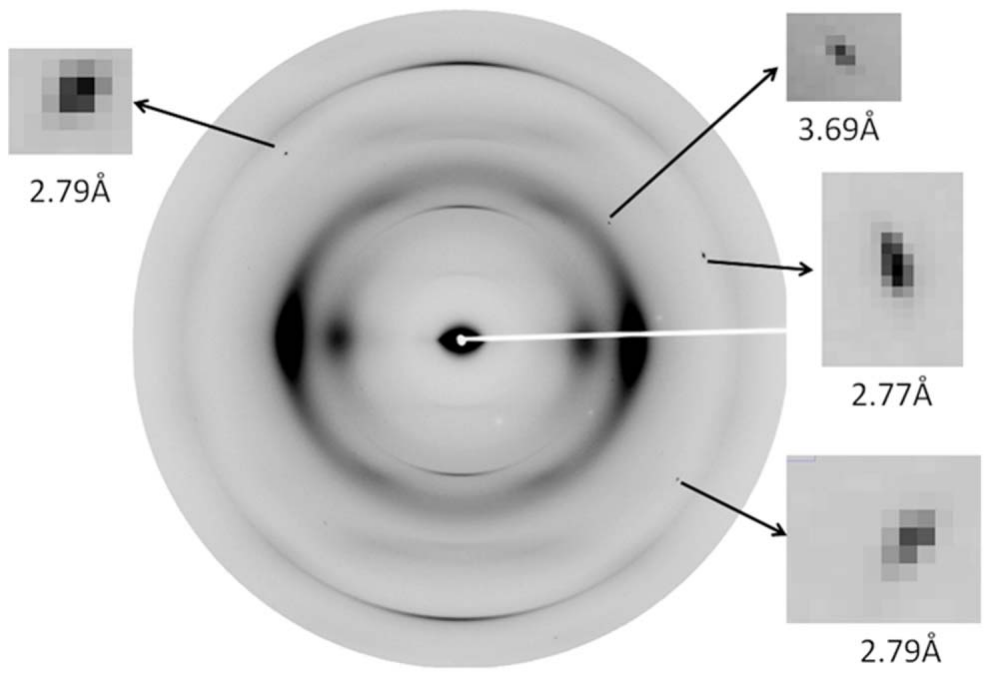
WAXS Pattern from vascular bundles of maize. This WAXS pattern shows diffraction characteristic of cellulose fibers and containing several Bragg reflections (magnified in the insets) that are due to diffraction from crystallites embedded in the material. At the point-to-point resolution of this pattern, taken at a sample to detector distance of 180 mm, little detail within the Bragg reflections can be discerned.

In order to survey the nature of x-ray scattering from the vascular bundles embedded in maize stalks, we collected wide angle x-ray scattering (WAXS) data using an 180 mm sample-to-detector distance at the BioCAT beam line at the Advanced Photon Source (APS). These patterns exhibited the characteristic intensity distribution expected for cellulose Iβ^9^ as well as occasional, very sharp reflections of uncertain origin (Fig. 1). These sharp reflections exhibited no apparent preferred orientation relative to the fiber axis of the stover. Thirty WAXS patterns from maize stalks were examined and the scattering angles of nearly 100 of these Bragg peaks were measured from these data sets. A comparison of the measured scattering angles with the d-spacings of reflections predicted from the crystal structures of naturally occurring minerals commonly observed in plant tissues concluded that almost all of these reflections could be indexed using the crystal lattices of calcium oxalate monohydrate (whewellite) and dihydrate (weddelite). The strongest reflections expected^10^,^11^ from whewellite (5.93, 3.64 and 2.98 Å) and weddelite (6.19, 4.42, 3.68 and 2.75 Å) were all observed in this survey, indicating that both crystal forms exist in these samples. The observation of Bragg peaks on most of these images shows that these microcrystals occur at a density that is typically high enough to find crystals in orientations that place a few strong reflections on the detector surface. For the x-ray beam size of 140 × 40 μm and assuming a sample thickness of ~1 mm the sample volume illuminated by x-rays in these experiments is ~6 × 10^6^ μm^3^. The calculations of three-dimensional diffraction patterns from the crystal structures^10^ of whewellite and weddelite showed that they produce very few strong reflections and, therefore, the probability of detecting diffraction from a specific randomly oriented crystal is small. If the mosaic spread of a crystal is assumed to be ~0.05° the crystal number density probably must exceed one crystal per 10^4^ μm^3^ sample volume to produce detectable Bragg peaks on the majority of WAXS images.

**Figure 2:**
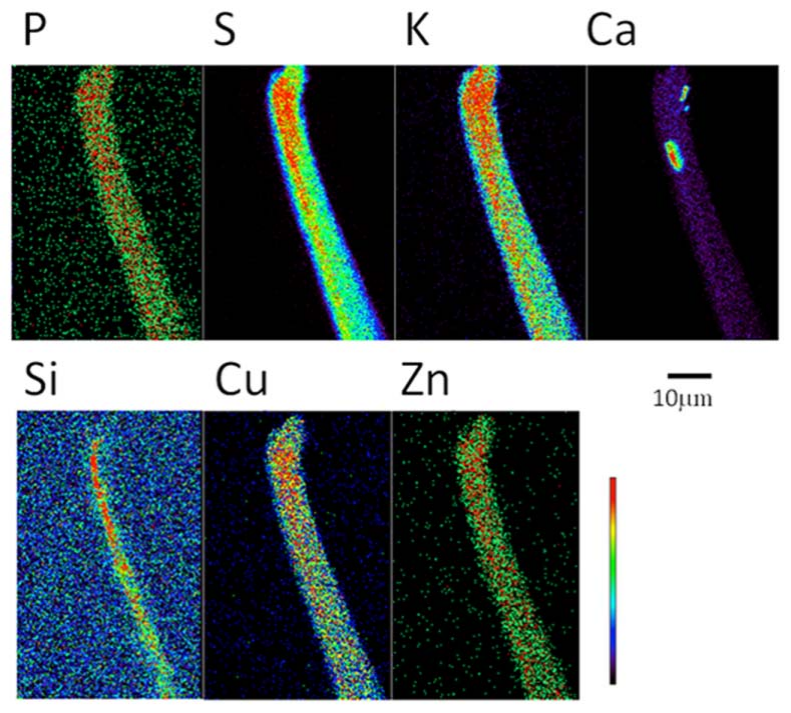
XFM images of maize. XFM images of a fiber cell teased from a sample of corn stover. Cell walls around the fiber cells are typically 10 microns in diameter and hollow. The distribution of most elements corresponds to nearly uniform distribution within the cell walls. The exceptions are calcium which exhibits several micron-sized calcium-rich inclusions, and silicon which appears to concentrate within the hollow interior of the fiber cells.

X-ray fluorescence microscopy (XFM) revealed occasional calcium-rich inclusions with linear dimensions of ~1 micron (Fig. 2). Since the samples used for the XFM analysis were from the same source as the stover used for the WAXS analysis these observations are consistent with the presence of calcium oxalate crystals. Although the resolution of the XFM images is relatively low, the inclusions appear to have well-defined shapes and edges. Silicon-rich inclusions were also observed, particularly in the hollow centers of fiber cells. However, Bragg peaks observed by WAXS could not be indexed on the basis of any known naturally occurring silicon-rich mineral. We concluded that the silicon-rich inclusions were either amorphous or constituted crystals sufficiently small that the scattering peaks were not detectable in the WAXS patterns.

**Figure 3:**
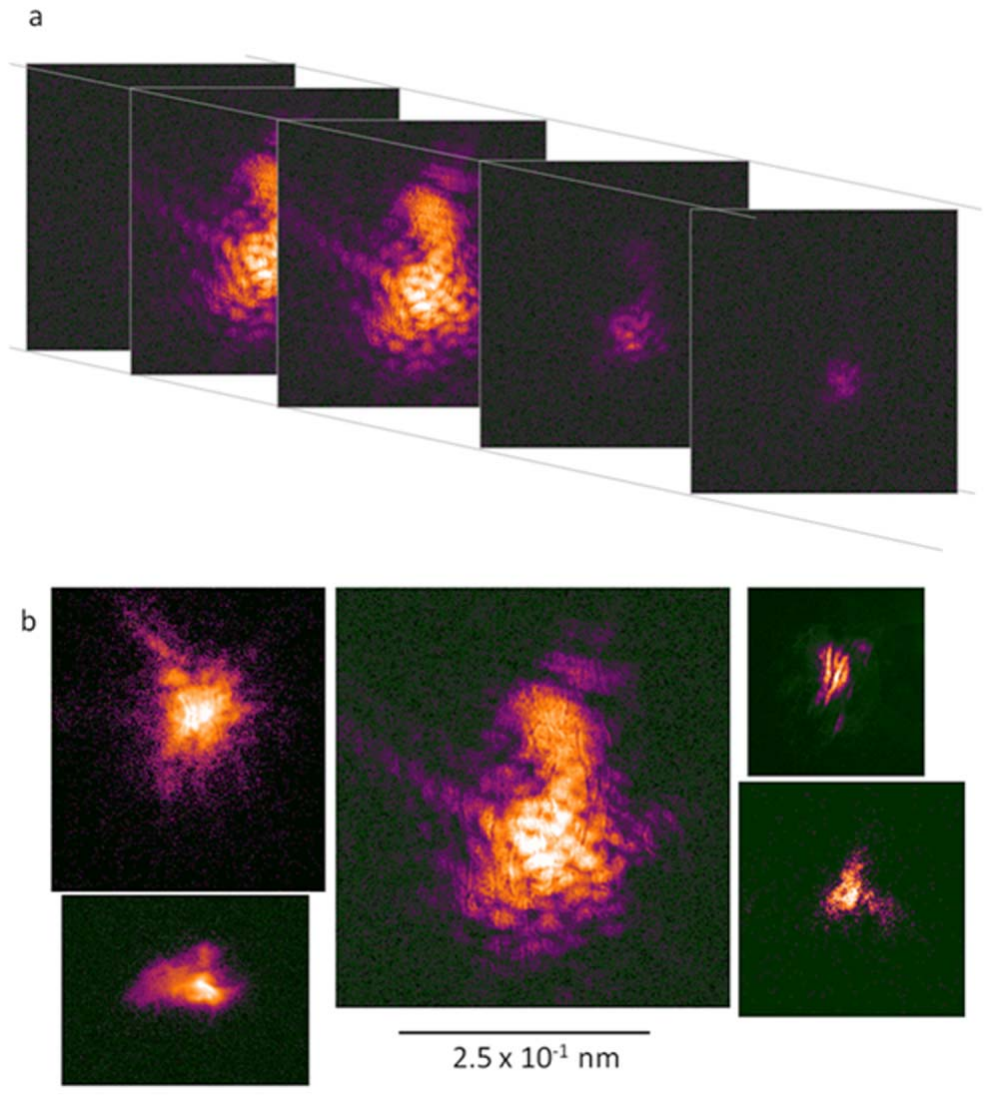
BCDI data. (a) Sections through a three-dimensional BCDI data set collected as a series of diffraction patterns in which each successive pattern is separated from the previous by a small, constant angular rotation of the detector, δθ. This illustration shows every fifth image taken as the detector rotated through the reflection. The stack of almost parallel images is then assembled into a three-dimensional data set that maps the detailed diffracted intensity around the position of a single Bragg reflection. (b) Central sections through five different Bragg peaks corresponding to the five reconstructions shown in Figure 4. The intensity measurement in all of the diffraction images are displayed on a logarithmic scale.

BCDI diffraction data were collected on a CCD detector mounted on a 6-circle diffractometer at beam line 34-ID-C at the Advanced Photon Source at Argonne National Laboratory using a beam size of approximately 2 μm^6^,^12^. Early observations at room temperature indicated that radiation damage to the surrounding organic material was leading to movement of the crystals in the beam. Consequently, all data were collected with the samples held at liquid nitrogen temperature. Vascular bundles teased from dried maize stalks were adhered to the cold finger of a cryostage with their fiber axes perpendicular to the incident x-ray beam. The individual vascular bundles, typically 1–3 mm in diameter, were broken from the bulk of the stalk and laid parallel to each other on the copper surface of the cold finger (see Supplementary Information, S1). The detector was placed at a scattering angle corresponding to a spacing of 3.65 Å where scattering from both forms of calcium oxalate can be observed. The samples were translated across the x-ray beam with the detector at 0.75 m from the sample in order to provide a relatively large field of view for detection of Bragg reflections. Once a Bragg reflection was observed, the detector was typically translated to either 1.0 m or 1.8 m from the sample in order to collect the data on the finest possible grid. By rotating the sample over small increments a three-dimensional data set was assembled as a set of two-dimensional slices that map the intensity through the reflection (Fig. 3a).

About a dozen data sets suitable for three-dimensional reconstruction were collected from vascular bundles harvested from dried maize stalks. Figure 3b shows a gallery of central sections through the diffuse speckles around Bragg peaks from five different samples. Measuring the distance from the center of a reflection to positions where the intensity disappears into the background suggests that the resolution achieved by the most strongly diffracting samples was ~50 nm in the dimensions that correspond to the detector surface, with more typical data sets achieving a resolution closer to ~100 nm. In the dimension corresponding to the sample rotation the maximum resolution is constrained by the extent of the rotation and proved lower, typically in the range of ~200–400 nm.

Image reconstructions of calcium oxalate crystals utilized these BCDI data sets and a local implementation of algorithms previously demonstrated to accurately determine the sizes and shapes of crystal objects^13^,^14^,^15^,^16^,^17^,^18^,^19^,^20^,^21^. The implementation was checked by reconstructing known objects from simulated data. The general approach involves the iteration of real-space object constraints, in which the image of the object under reconstruction is retained only within the confines of a support volume, and a reciprocal-space step, in which phase angles from the back transform of this modified image are recombined with the experimentally determined diffraction amplitudes.

**Figure 4:**
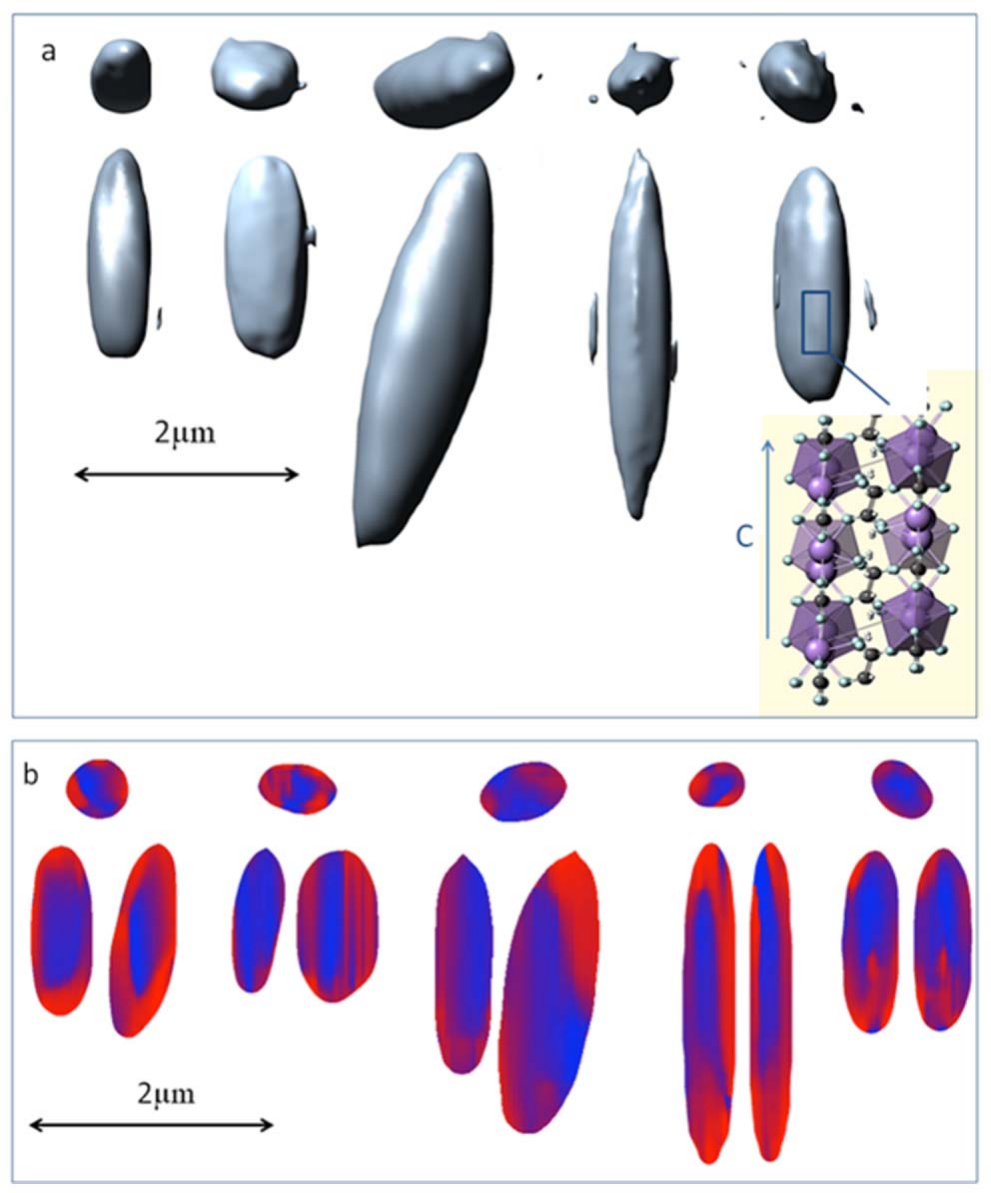
Crystal reconstructions from BCDI data. (a) Top and side views of isosurface renderings of the absolute-valued electron density for five crystals reconstructed from BCDI data. The threshold value of the surface contour was set to make visible the largest extraneous noise feature in the map. Noise and spurious detail in the reconstructed images were suppressed by local smoothing of the density over volumes with dimensions approximately equal to the resolution of the data. The inset image depicting the atomic structure of the whewellite crystal was obtained from the *Mineralogy Database* (http://webmineral.com/data/Whewellite.shtml)^28^ with the c-axis running in the vertical direction. (b) Orthogonal slices showing phase maps through the centers of the corresponding crystals shown in (a). The top row shows the set of cross-sections as viewed down the c-axis and the bottom row shows the two corresponding longitudinal sections for each reconstruction. The color table rotates from blue (0 radians), through red (π radians) and reverts to blue (2π radians). The values of phase angles outside the volume of the final support have been eliminated for clarity. The density isosurfaces were rendered using the *Chimera* program^29^ and the phase diagrams were rendered with the *ImageJ* software^30^.

Renderings of crystals reconstructed from the BCDI data (Fig. 4a) indicate that they range in length from 2–5 μm with cross sections 0.5–1 μm across. The images are oriented identically in the frame of reference of the detector with the plane of the detector horizontal and perpendicular to the plane of the images. The two longest crystals have a symmetric appearance with flattened cross sections and pointed ends whereas the other three examples are ~2 μm in length and have blunt ends. Some aspects of each crystal show indications of the presence of flat faces. Disordered material or crystalline material oriented in such a way that it does not scatter into the Bragg peak being used for the reconstruction will not contribute to the reconstruction and result in a void. Consequently, these images correspond to ordered crystalline domains giving rise to scattering in the observed Bragg reflection.

The crystals, reconstructed on the basis of a Bragg peak at ~3.65 Å spacing, could be either calcium oxalate monohydrate or dihydrate since the (0 4 0) peak of whewellite and the (0 0 2) peak of weddelite both scatter very near that angle. However, an argument based on the known crystal symmetries for these two crystal forms shows that all of these reconstructions correspond to the monohydrate form. First, the 3.65 Å periodicity is fixed in a direction perpendicular to the long axis of these crystals. In weddelite this d-spacing corresponds to the (0 0 2) reflection so the periodicity is placed on the crystal c-axis. Since weddelite crystallizes in a tetragonal space group the a- and b-cell axes are equal and the (1 0 0) and (0 1 0) directions are equivalent. Consequently, the required orientation would place one of these two equivalent axes along the long axis of the reconstructed crystal and the other perpendicular to the long axis. In contrast to this contradictory situation, whewellite crystals are frequently observed as having long, thin shapes with pointed ends. These crystals are referred to as ‘raphides’ and the c-axis of whewellite has been shown to correspond with the long axes of raphides^11^. These observations indicate that the crystals that we have reconstructed are whewellite.

The x-ray scattering patterns about the Bragg peaks analyzed in this study are characterized by strikingly asymmetric intensity distributions that sometimes contain a distinct substructure of streaks and an ill-defined central maximum (Fig. 3). Although the overlapping peaks that occur in merohedrally twinned crystals may also lead to complicated intensity distributions^22^, asymmetry in the intensity distribution around the position of a Bragg reflection generally requires that the real-space object be considered as a complex rather than purely real electron density function^8^,^7^. A complex-valued electron density may be represented by a function that contains both a magnitude and phase angle component. The magnitude of this function represents the physical electron density and, as in the more conventional case of a purely real electron density function, the absolute value of this term defines the shape and extent of the object. The phase component measures the local deformation of the crystal lattice and variations in phase across the object correspond to shifts of diffracting units within the object relative to the periodicity of the ideal crystal. Consequently, a phase map calculated over the object volume may be used to identify positional variations in the crystal. A phase shift of π radians is indicative of a part of the object shifted by half a unit cell from its ideal position. For example, in BCDI analysis involving manufactured specimens of very simple crystals this representation has been used to map strain fields across the crystal^8^,^23^. Phase angles are sensitive to positional shifts on length scales less than the unit cell of the crystal and, therefore, probe changes in the crystal periodicity on an angstrom length scale.

Examination of the distribution of the phase angle component in the complex electron density reveals sharp separation of the crystal into domains of relatively uniform phase (Fig. 4b). This arrangement of domains is characterized by a phase shift exceeding π/2 radians, indicative of over a quarter-unit cell shift in one part of the crystal relative to another. Most of these reconstructions show a central crystal core surrounded by a shell with shifted phase. The presence of an intermediate structural layer of sub-resolution dimensions (~50 nm) at the domain boundaries cannot be ruled out although there is no evidence for a component of this type. AFM studies of whewellite crystal growth identify the crystal face associated with the crystal a-axis as the slowest growing face and prone to a variety of crystal imperfections^24^. The surface of this face is not atomically smooth since it is composed of oxalate ions in two different orientations and a mismatch of crystal blocks at this interface (as in a grain boundary) would result in a significant phase shift across the crystal. In addition, when viewed down the b-axis the whewellite crystal contains planes of calcium and oxalate parallel to the c-axis in which a significant slippage would result in relatively little disruption in the organization of intermolecular bonds (inset in Fig. 4a).

The x-ray scattering at a particular Bragg reflection (in this case the (0 4 0) reflection) is associated with a specific orientation of the reflecting planes in the crystal relative to the incident x-ray beam. For this reason the lattice orientation in the crystal reconstructions from the BCDI data is fixed by the selection of Bragg reflection for analysis. For the largest crystal in Fig. 4a the c-axis is vertical although this orientation is tilted by about 20° from the long axis of the crystal. The phase distribution in this crystal (Fig. 4b) indicates that the dislocation plane is parallel to the c-axis of the crystal lattice rather than the long axis of the crystal. This suggests the dislocation is not uniquely linked to the crystal habit but is defined by the orientation of the underlying lattice.

## Discussion

Plants control the size and shape of biomineral inclusions^25^. Although capable of taking on several distinct habits, whewellite most often takes on the elongated shape known as a raphide. Raphides in *Araceae* are elongated, frequently pointed, and accommodate grooves along their long axes^3^. The combination of sharp ends and adsorbed proteases produces toxic responses when plants harboring raphides are ingested. The sharp ends of these crystals appear to be developed so as puncture cells and introduce irritant into organisms ingesting the material^2^,^3^. The observation here of crystal particles organized into distinct crystal domains related by a large dislocation of the crystal unit cell is suggestive of a new level of biological control in the deposition of biominerals. The function of this dislocation is not clear but may contribute to the formation of the sharp ends of raphides.

Cellulosic ethanol production technologies make use of the whole plant (rather than just the corn kernels) and are, therefore, potentially appealing to the biofuel industry as a more efficient means for generating ethanol than standard fermentation techniques. Calcium oxalate crystals are the prevalent mineral deposit in higher plants but have usually been identified in plant leaves and have not, to our knowledge, been previously reported in maize stalks. Characterization of the chemical composition and sizes of calcium oxalate particles embedded within maize stalks could be of value in the development of industrial processes for their breakdown and subsequent purification to ethanol. Additional techniques, such as x-ray micro-tomography, are able to provide the number density of micro crystals in plant tissues^26^.

These results demonstrate the level of detailed characterization that can be accomplished in the study of microcrystals embedded in an opaque, heterogeneous material; identification of the mineral composition of the inclusions; their size and shape; the orientation of the crystal lattice within the crystal; the form and nature of dislocations. The microscale architecture of heterogeneous materials, natural or synthetic, is central to providing the extraordinary properties that make them increasing popular for a broad range of applications. BCDI represents a novel, non-invasive approach to characterization of this architecture at a level of detail difficult to achieve by other methods and essential as a basis for future materials design.

## Methods

WAXS data were collected at beam lines 18-ID (BioCAT) and 23-ID-B (GMCA CAT) at the Advanced Photon Source (APS), Argonne National Laboratory, using a sample to detector distance of approximately 180 mm and a MAR165 (at BioCAT) or a sample to detector distance of 300 mm and a MAR300 detector (GMCA CAT). XFM images were collected at beam line 2-ID-E at the APS as previously described^27^ using 10 keV incident energy, 300 nm step size and 50 ms dwell time per pixel in fly scan mode (continuous scan motion in sample x direction). Fluorescence spectra per pixel were detected by a four element Si drift detector (Vortex-ME4 by SII NanoTechnology).

BCDI data sets were collected at beam line 34-ID-C at the Advanced Photon Source using instrumentation as previously described^8^. Briefly, data collection involved scanning the sample past the x-ray beam while monitoring for the presence of Bragg peaks with a relatively low (~0.75 m) sample to detector distance. Once observed, the sample to detector distance was usually increased to either 1.0 or 1.8 m for data collection at high point-to-point resolution. Crystal size was assessed by scanning across the sample in two directions. Full data sets were collected only from crystals that were completely irradiated by the incident beam. The data collection strategy focused on crystals oriented so that their long axis was approximately parallel to the incident x-ray beam making possible reconstruction of crystals with long axes substantially longer than the diameter of the incident beam.

The detector is able to sample a reciprocal space interval of Δq_xy_ = 2π Δp_xy_/λL, where Δp_xy_ is the pixel size of the detector, L is the sample to detector distance and λ is the wavelength of the incident x-ray beam. For the data sets used for image reconstruction the pixel size of the detector was 22.5 μm, the crystal to detector distances were 0.75 m, 1.0 m and 1.8 m and the x-ray wavelength varied from 1.392 Å to 1.409 Å (see Supplementary Information, S2). With these parameters the reciprocal space samplings, Δq_xy_, for data recorded on the detector were in the range 0.0056–0.0010 nm^−1^. All data sets were collected from a reflection characterized by a scattering angle of ~23° (corresponding to a spacing of ~3.65 Å) and this reflection was scanned by rotating the sample in increments ranging from 0.0017° to 0.0047°. By selecting an appropriate value for the rotation increment, the reciprocal space sampling, Δq_z_, between consecutive, almost parallel images may be approximately matched to the reciprocal space sampling in the detector plane, Δq_xy_.

The reconstruction process was initiated by associating experimentally measured amplitudes with randomly selected phase angles and computing the Fourier transform. The real-space modification step employed both error reduction (ER) and hybrid input-output (HIO) algorithms^14^,^15^ for changing the density outside the support volume. The initial support volume was generated by smoothing the autocorrelation function of the object (*i.e.* the Fourier transform of the intensities) and taking grid points where this function exceeded 4% of the function maximum as defining the support^20^. The full image reconstruction protocol required several macrocycles with each macrocycle including 10 iterations of ER, 50 iterations of HIO and 50–100 further iterations of ER. After each macrocycle the support volume was updated using the shrink wrap procedure^20^, which allows the support volume to evolve towards the shape and size of object that is being reconstructed. Convergence of this process was measured by a χ^2^ statistic that compared the amplitudes computed from the Fourier back-transform of real-space arrays modified by the ER or HIO methods with experimentally determined amplitudes^6^. After 5–10 macrocycles stable values for the support volumes and χ^2^ statistics were obtained (see Supplementary Information, S3).

The uniqueness of these solutions to the image reconstruction problem was checked by performing replicate runs for each data set in which different sets of random phases were used to initiate each run. Compensation was made for the known ambiguities, involving translations and inversions of the reconstruction solutions^15^, when comparing parallel reconstruction runs. These tests initially showed that the shapes and sizes of the crystal reconstructions were consistently determined from the absolute values of the final electron densities but the maps of the phase angle component of the complex electron density functions were not necessarily unique at this level of data accuracy. In order to obtain more accurate input data for the reconstruction process, the values of neighboring intensity measurements were averaged together. *i.e.* the reciprocal-space data sampling was slightly reduced in exchange for the greater statistical accuracy of the merged measurements. Reconstructions performed with this preprocessed data were characterized by lower final χ^2^ values and a much more consistent appearance in the maps of the phase angles associated with the complex electron density. Calculations from simulated scattering patterns from various objects in which the scattering center was miss-set or in which random counting noise was introduced into the intensity distribution were performed to assess the impact of these types of error on the resulting complex electron density maps. In neither case did the resulting phase maps contain features that resembled the distinctive patterns of phase domain separation that are observed in the reconstructions from these experimental BCDI data. Also, in test calculations where the starting electron density distributions (calculated from random phases) were set to purely real values, the expected complex electron density distributions still emerged from the reconstruction process, further demonstrating that the complex electron density distributions we have obtained are an inherent consequence of the asymmetric intensity distributions observed around the Bragg scattering positions.

The BCDI reconstructions were initially calculated on a 3D grid defined by two dimensions in the plane of the detector (typically 200–300 pixels in side length) and a third dimension defined by the sample rocking angle. The resulting maps of the complex electron density were subsequently transformed to a cubic Cartesian coordinate system for display^8^.

## Author Contributions

J.B. carried out the final computational analyses and interpretations and wrote the manuscript; J.L. collected most of the data and carried out extensive preliminary data analyses; R.H. designed the experiments, was involved in all the data collection and advised of all aspects of the project; H.I. was involved in analyses of the WAXS data and interpretation of the BCDI data; S.C.G. and S.V. collected and analyzed the XFM data; I.R. designed the experimental apparatus, was involved in data collection and advised on other aspects of the project; L.M. conceived the project, coordinated all aspects and wrote the manuscript.

## Supplementary Material

Supplementary Information Supplementary Information for Three-dimensional Imaging of Crystalline Inclusions Embedded in Intact Maize Stalks
