# Supplementary material for: Three-dimensional Imaging of Crystalline Inclusions Embedded in Intact Maize Stalks
Source: Sci Rep. 2013 Oct 3;3:2843. doi: 10.1038/srep02843 (PMC3789445; doi:10.1038/srep02843)
Supplement: Supplementary Information — for Three-dimensional Imaging of Crystalline Inclusions Embedded in Intact Maize Stalks [file srep02843-s1.pdf]

Supplementary Information for

Three-dimensional Imaging of Crystalline Inclusions Embedded in Intact Maize Stalks

John Badger[1], Jyotsana Lal[2], Ross Harder[2], Hideyo Inouye[3], S. Charlotte Gleber[2], Stefan Vogt[2], Ian Robinson[4,5] and Lee Makowski[3*]

1. DeltaG Technologies, San Diego, California 92122, USA

2. X-ray Science Division, Advanced Photon Source, Argonne National Laboratory, Argonne, Illinois 60439, USA

3. Dept. of Electrical and Computer Engineering, Northeastern University, Boston, Massachusetts 02115, USA

4. London Center for Nanotechnology, University College London, London WC1E 6BT, UK

5. Research Complex at Harwell, Oxford, OX11 0FA, UK

* Correspondence to makowski@ece.neu.edu

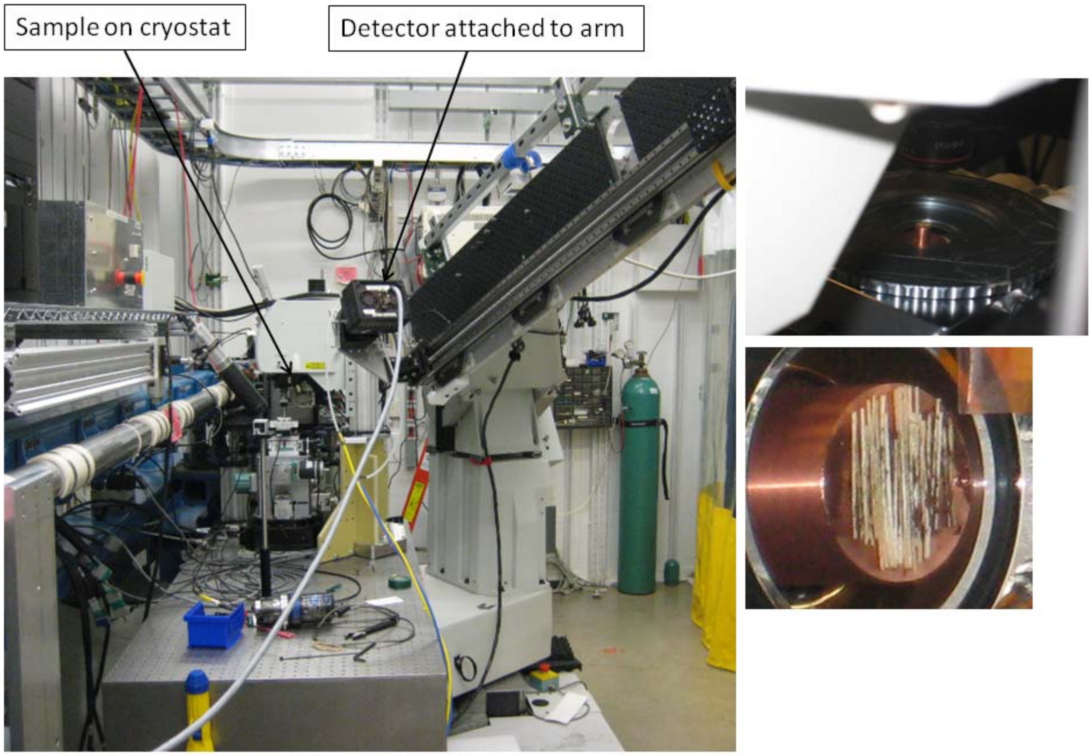

S1 | Experimental setup for the collection of BCDI reconstruction data. The photograph on the left shows the position of the cryostat and the detector attached to a variable length arm. In this view the x-ray beam is directed almost directly from the sample towards the detector. The image on the top right is a close-up of the cryostat. The image on the bottom right shows the attachment of vascular bundles to the cryostat.

Sample ID	Wavelength (Å)	Sample-Detector distance (m)	δ (°)	γ (°)	No. images	Scan increment (°)
1209_40	1.396	1.00	23.005	1.504	85	0.0025
1209_100	1.392	1.00	23.054	0.649	31	0.0047
409_150	1.403	1.80	22.998	-0.731	41	0.0025
409_222	1.403	1.80	22.501	-0.005	31	0.0017
711_80	1.409	0.75	2.003	21.849	51	0.0040

S2 | Experimental parameters for the BCDI reconstruction data. The δ and γ angles correspond to the two circles on a 6-circle diffractometer that define the x-ray scattering angle, 2Θ. The number of images is the number of slices collected in each data set by successive rotations through small angular increments, δΘ.

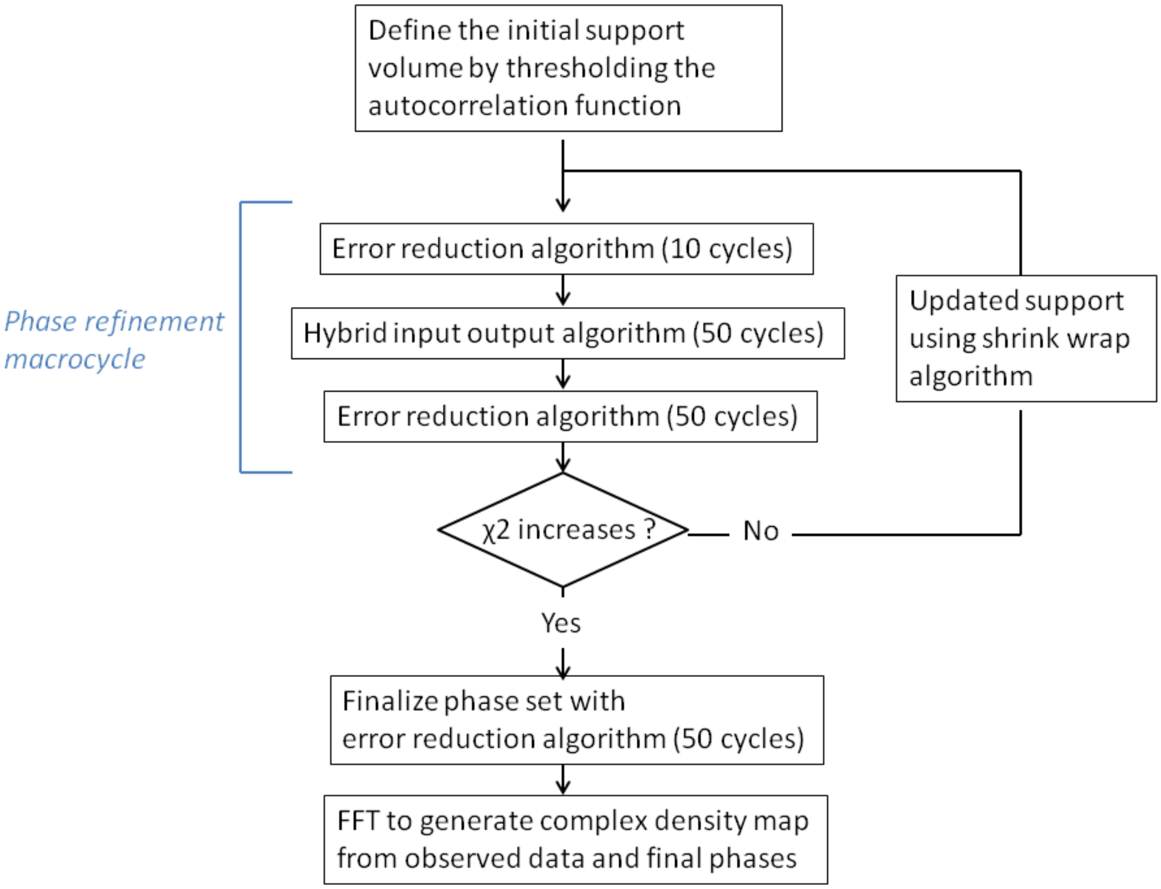

S3 | Flow chart of the iterative phase determination process for image reconstruction from BCDI data. After generating an initial estimate for the object support from the autocorrelation function[1] a single macrocycle for image improvement employs iterative cycles of error reduction and hybrid input-output algorithms [2-4]. Following a single macrocycle, the shrink wrap algorithm[1] is used to obtain an improved estimate for the support volume. Progressively reducing the dimension of the convolution function used in the shrink wrap algorithm typically results in a smaller and more detailed support. The $\chi 2$ statistic, comparing observed and calculated structure factor amplitudes, is used as the criteria for managing the dimension of the convolution function used by the shrink wrap algorithm.

1. Marchesini, S. *et al.* X-ray image reconstruction from a diffraction pattern alone. *Phys. Rev. B*. **68**, 14010-1 - 14010-5 (2003).

2. R.W. Gerchberg & Saxton, W.O. A practical algorithm for the determination of the phase from image and diffraction plane pictures. *Optik* **35**, 237-246 (1972).

3. Fienup, J.R. Phase retrieval algorithms: a comparison. *Applied Optics*. **21**, 2758-2769 (1982).

4. Fienup, J.R. Reconstruction of a complex-valued object from the modulus of its Fourier Transform using a support constraint. *J. Opt. Soc. Am. A.* **4**, 118-123 (1987).
